# Antibody development to identify components of IIS and mTOR signaling pathways in lepidopteran species, a set of non-model insects

**DOI:** 10.17912/micropub.biology.000755

**Published:** 2023-02-16

**Authors:** Alissa R Armstrong, Carol L Boggs

**Affiliations:** 1 Biological Sciences, University of South Carolina, Columbia, South Carolina, United States; 2 School of the Earth, Ocean & Environment and Department of Biological Sciences, University of South Carolina, Columbia, South Carolina, United States

## Abstract

Nutritional stress impacts many insect species that have differing reproductive strategies and life histories, yet it is unclear how nutrient-sensing signaling pathways mediate tissue-specific responses to changes in dietary input. In *Drosophila melanogaster* , insulin/insulin-like growth factor (IIS) and mTOR-mediated signaling within adipocytes regulates oogenesis. To facilitate comparative study of nutrient-sensing pathway activity in the fat body, we developed antibodies to assess IIS (anti-FOXO) and mTOR signaling (anti-TOR) across three nymphalid species (Lepidoptera). By optimizing whole-mount fat body immunostaining, we find FOXO nuclear enrichment in adult adipocytes, like that observed in *Drosophila* . Additionally, we show a previously uncharacterized TOR localization pattern in the fat body.

**Figure 1. Antibodies generated using FOXO and mTOR lepidopteran epitopes are immunoreactive in lepidopteran fat body:**
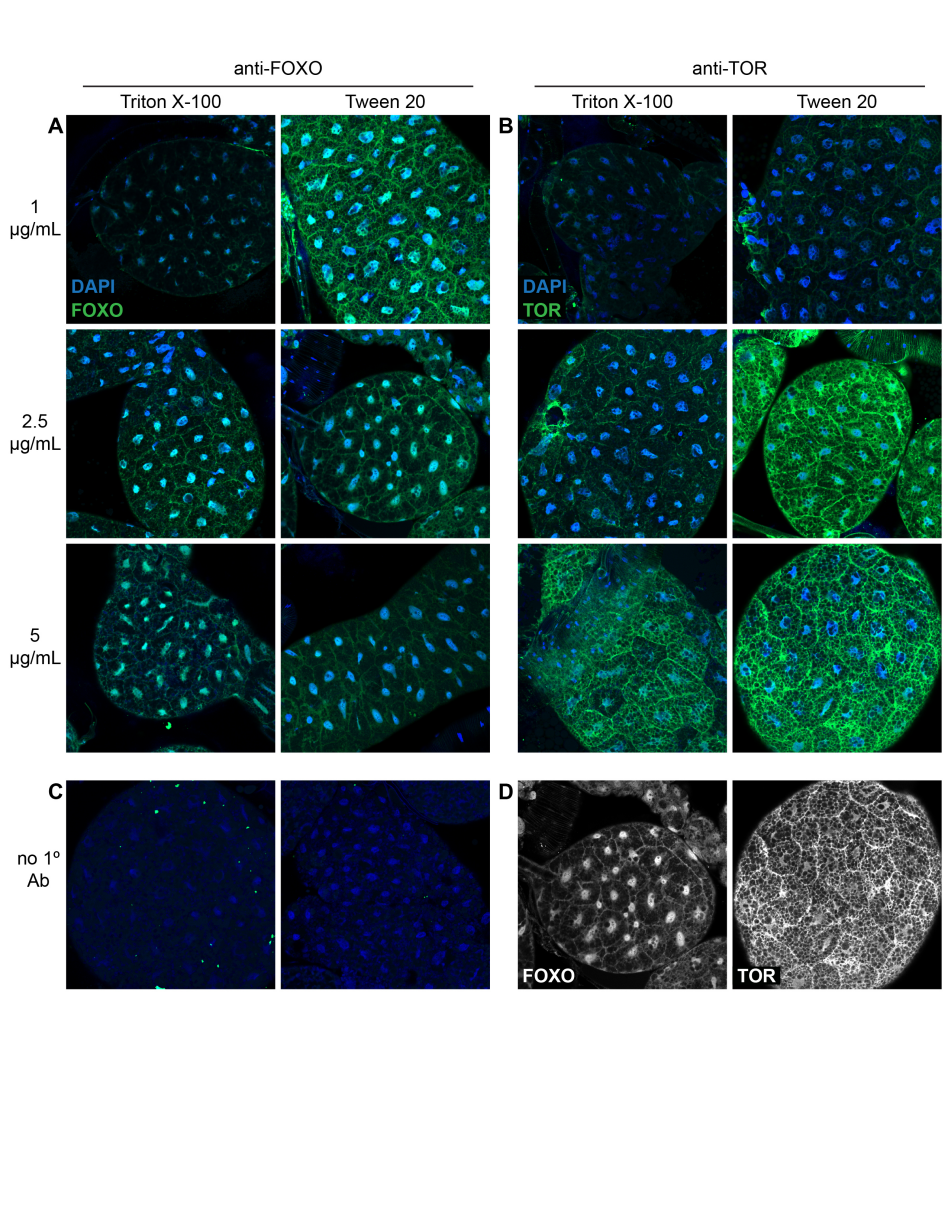
Fat body tissue from *Heliconius charithonia* was labeled with increasing concentrations of FOXO (A) or mTOR (B) antibodies using either Triton X-100 or Tween 20 as the detergent in wash solutions (green, antibody; blue, DAPI). Fat body tissue incubated without primary antibody shows that there is no non-specific binding of the secondary antibody (C). Single channel images of the antibody concentration (anti-FOXO, 2.5 μg/mL; anti-mTOR, 5 μg/mL) and detergent (Tween 20) combination that showed the best immunoreactivity (D).

## Description

Resource allocation is inextricably linked to tradeoffs between life-history traits, allowing organisms to adapt to a dynamic environment (Ng’oma et al., 2017). Nutritional control of the tradeoff between survival and reproduction is one of the best-characterized examples of this phenomenon (Schwenke et al., 2016; Krittika and Yadav, 2019) and has been described for a variety of organisms, including insects. In several insect species, nutrients are reallocated from the energy intensive process of oogenesis to support somatic tissue maintenance during nutrient deprivation (Boggs and Ross 1993; Zera and Harshman, 2001). This ensures that organisms survive beyond suboptimal nutritional conditions and do not waste resources on reproduction when the likelihood of offspring survival is low. While it is likely that multiple tissues sense and respond to nutrient input, we are just beginning to understand how inter-organ communication mediates whole organism responses in the heavily studied insect model, *Drosophila melanogaster* . For example, nutrient sensing pathways, like IIS, mTOR, and the amino acid response (AAR) pathway, function within adult *Drosophila* adipose tissue to control distinct stages of oogenesis (Armstrong et al., 2014; Armstrong and Drummond-Barbosa, 2018). However, insects as a group that potentially includes 5.5 million species (Stork, 2018) have diverse life histories. For example, female reproductive strategies are influenced by differences in ovarian dynamics (Dunlap-Pianka et al., 1977), egg carbon:nitrogen ratios (O’Brien et al., 2004), and ovariole numbers (Church et al., 2021). Insects also vary in distribution of the adipose tissue within the body, which could potentially affect inter-organ communication. *Drosophila* is therefore an excellent model for one such suite of traits but is not representative of all insects. We must develop additional diverse insect models in order to understand the variety of mechanisms controlling life history responses to dietary stress across insects.

Given that nutrient sensing signaling pathways control the response of insect fat body to changes in dietary input, which in turn affects allocation to egg production and other life history functions (Smykal and Raikhel, 2015; Johnson et al., 2014), we are interested in comparing the operation of these pathways and their outcomes among *Drosophila melanogaster* and various butterfly species within the Nymphalidae (Lepidoptera). These species differ in fat body distribution (Haunerland and Shirk, 1995), ovarian dynamics (McLaughlin and Bratu, 2015; Dunlap-Pianka et al.,1977), egg C:N ratio (Min et al., 2006; O’Brien et al.,2004) and completeness of the adult diet (Piper, 2017; Gilbert, 1972; O’Brien et al, 2004).

The response to dietary stress of the IIS pathway has been well characterized in *Drosophila.* Upon insulin receptor activation, Akt, the main effector kinase, acts on several targets to regulate cellular metabolism, survival, proliferation, and growth as well as reproduction (Grewal, 2009; Partridge et al., 2011; Manning and Cantley, 2007). When phosphorylated by Akt, the transcription factor FOXO is restricted from the nucleus, where it controls expression of genes that mediate the negative effects of reduced IIS (Manning and Toker, 2017). In addition, Akt indirectly activates the mTOR kinase by inhibiting the negative regulatory complex of mTOR, Tsc1/Tsc2 (Potter et al., 2002). The mTOR kinase can also be activated by the Ragulator complex which senses amino acid status (Dibble and Cantley, 2015). In both cases, mTOR activation happens at the lysosome.

To gain a better understanding of how IIS functions within lepidopteran fat tissue, we want to visualize changes in localization of FOXO and mTOR. One approach to tracking localization of these components is to use species-specific antibodies for immunocytochemistry. While the required antibodies for FOXO and mTOR are available for *Drosophila,* the transcriptomes for our non-model lepidopteran species have very limited similarity to those of *Drosophila melanogaster* (<68% for *Heliconius charithonia* and *Agraulis vanillae* for FOXO; 48% for *H. charithonia* and 61% for *A. vanillae* for mTOR). Therefore, the likelihood of successful use of *D. melanogaster* antibodies in the lepidopteran species is low. In contrast, our identified FOXO and TOR transcriptomes for the three lepidopteran species had high percent similarity (> 95%), indicating likely success in developing an antibody for each protein that would work in all three butterfly species.

In this report, we describe the development of Lepidoptera-specific antibodies for FOXO and mTOR to track changes in localization of FOXO and mTOR. To generate antibodies for FOXO and TOR in *Agraulis vanillae* (Lepidoptera: Nymphalidae) and *Heliconius charithonia* (Lepidoptera: Nymphalidae), we first determined the protein sequences for those genes for each species. We downloaded amino acid sequences for *Bombyx mori* (Lepidoptera: Bombycidae) and *Heliconius melpomene melpomene* (Lepidoptera: Nymphalidae) for FOXO and TOR from uniprot.org. We then used GeneiousPrime to generate transcriptome sequences for these proteins. We blasted these sequences against *A. vanillae* and *H. charithonia* transcriptomes to locate the sequences for each gene for these species. Transcriptome data for *H. charithonia* came from Catalan et al (2018), and for *A. vanillae* from Hanly et al (2019). Additionally, we used transcriptome and amino acid sequences for *Speyeria mormonia* (Cicconardia pers comm; Cicconardia et al 2022), because we are interested in experiments with that species in the future. These RNA sequences were converted to amino acid sequences for determination of epitopes joint among the species. Epitope identification and antibody production were done by Genscript. Rabbits were used for polyclonal antibody production. We also used GeneiousPrime to compare the similarity of FOXO and TOR RNA sequences among our three species, as well as with *Drosophila melanogaster* (Diptera: Drosophiladae).

We used a standard whole-mount immunostaining protocol to test immunoreactivity of these antibodies in lepidopteran fat body. We varied antibody concentration (1 μg/mL, 2.5 μg/ml, and 5 μg/mL) and permeabilization solution detergent (Triton X-100 versus Tween 20). We find that both antibodies show immunoreactivity in lepidopteran fat body at all three concentrations with varying robustness (Fig. 1) in all three butterfly species tested. For both antibodies, higher concentrations produced stronger signal (Fig 1A, B compare 1 μg/mL images to 5 μg/mL images). FOXO immunoreactivity showed enrichment in the nucleus with diffuse labeling in the cytoplasm (Fig. 1A). Since FOXO translocation to the nucleus results from reduced IIS pathway activity, this data suggests that the butterflies from which the fat body originated may have been under nutrient stress. Alternatively, lepidopterans may have a higher baseline of FOXO-dependent gene expression than *Drosophila* since adipocytes in fruit flies from nutrient-rich conditions show low-nearly undetectable levels of FOXO nuclear localization (Armstrong and Drummond-Barbosa, 2018). The majority of mTOR immunoreactivity shows diffuse cytoplasmic localization with slight enrichment at cell-cell junctions (Fig. 1B). Under nutrient replete conditions, mTOR localizes to lysosomes where it can be activated by amino acids (Sancak et al., 2010). Thus, future studies will assess if the mTOR staining pattern in butterfly adipocytes colocalizes with lysosomal markers. For both FOXO and mTOR immunolabeling, we are confident that the observed staining patterns are specific since there is no fluorescence in samples incubated without primary antibody (Fig. 1C). Moreover, the nuclear localization of FOXO is quite different from the cytoplasmic localization of mTOR (Fig. 1D). There is stronger fluorescence intensity at the lowest antibody concentration for tissue treated with Tween 20 than that treated with Triton X-100 (Fig. 1A, B). At higher antibody concentrations, detergent type is not a major factor in immunostaining quality. Taken together, we achieve the best signal-to-noise ratio for both antibodies when using Tween 20 as the detergent and 2.5 μg/mL of anti-FOXO and 5 μg/mL of anti-mTOR (Fig. 1D).

In the future, we will further optimize the immunostaining protocol by modifying additional steps in the protocol, such as fixative type and fixation time. This work demonstrates the feasibility of using these newly developed lepidopteran antibodies to answer questions like how generalizable are nutrient sensing mechanisms in *Drosophila* to other insects that differ in their fat body distribution, mode of ovarian function (e.g. stem cell-supported versus not), and adult diet macronutrient composition?

## Methods

*Butterfly collection*

*Agraulis vanillae* females were collected in Sunrise, Florida in January 2022. *Heliconius charithonia* females came from Davie, Florida and south Texas in January 2022 and fall 2021 respectively.

Antigens

FOXO: 

MSLPRGGSYQSPWSSQTALSELEGAMGEFEPVGELAEVGFEPQTRARSNTWPQPRPENYVDAEDPGSKKNSNQNLSGAPPLPTSATTKKNSSRRNAWGNLSYADLITQAITSSQEKRLTLSQIYEWMVQNVPYFKDKGDSNSSAGWKNSIRHNLSLHNRFMRVQNEGTGKSSWWMINPDAKPGKSVRRRALSMETSKTEKRRGRVKKKPDALRNGVTADATPSPSSSISESLDIFPDSPIHSSFQLSPDFRQRASSNASSCSRLSPIPSLIPTEPEWSTDYTPSGDFASTSDFSPADYTQDQDLAGSLADSMKLHGADPFLNTYVPTTSSSSSGGSYRFTYGACPRHPHGGCACAPLYPAHPAHPTHSHQHSLDHFVRPPPPADPADIMQTENSQTQMVATSDATLMNGGIMVQTGPMGPTTVMGQIMGALNTGLSEDLNIEALEHSFDCNVDEVIRHELSMDGTLDFNFPQQNTAMAAEAESQFRAPSAPVPTTLSGGGASQTPYTVAPSWVH.

TOR: 

MYMFLLKGHEDLRQDERVMQLFGLVNTLLQADPDTFRRDLVIQRYAVIPLSTNSGLIGWVPHCDTLHSLIKDYREKRKILLNIEHRIMQRMASDLEKLMLMQKVEVFEHALEHTAGDDLAKLLWLKSPSSEVWFERRTNYTRSLAVMSMVGYILGLGDRHPSNIMLDRVTGKFLHIDFGDCFEVAVTRDKFPEKIPFRLTRMLINAMEVTGIEGTYRRTCESVMEVLHRHRDSVMAVLEAFVYDPLLNWRLIDAGRRAGAPDDDGDASPQRPDDAPAETNLNKRALAIVNRVRDKLTGRDFTHIEENSVSVQRQVDLLIQQATSNENLCQCYVGWCPFW.

*Whole-mount adipose tissue immunostaining*

Females were sacrificed by crushing the ventral nerve and their abdomens were immediately dissected in cold phosphate buffered saline (PBS) to obtain fat body. Fat bodies were placed in 1.5ml microfuge tubes that were previously coated with 3% bovine serum albumin (BSA). Fat bodies were fixed in 1ml of 4% paraformaldehyde diluted in cold PBS (PFA) for 30 minutes at room temperature, rocking on a nutator. The PFA was then removed, and samples were rinsed twice with 1ml of PBS containing either 0.1% Triton X-100 (PBTX) or Tween 20 (PBTW) as a detergent. After rinsing, samples were washed three times with 1ml of 0.1% PBTX or PBTW, rocking each time for 15min on a nutator at room temperature. After the last wash, samples were incubated in 1ml of a blocking solution (0.1% PBTX or PBTW in 5% BSA) overnight at room temperature on a nutator. Samples were incubated in the indicated primary antibody concentration, diluted in blocking solution, overnight at room temperature. After washing three times for 15 minutes with PBTX or PBTW, samples were incubated in goat anti-rabbit AlexaFluor 488 secondary antibody diluted 1:250 in blocking solution and protected from light by covering with foil. Samples were then washed three times for 15 minutes with 0.1% PBTX or PBTW, protected from light. Following removal of wash solution, two drops of VectaShield containing DAPI were added. Samples were stored at 4 ^ο^ C until mounted on slides.

## Reagents

1X PBS – made from 10X PBS (Thermo Scientific, J75889-K8)

3% BSA – made from 30% BSA (VWR, K719-500ML)

4% PFA – made from 16% PFA (Electron Microscopy Sciences, 15710)

Triton X-100 - VWR, 0694-1L

Tween 20 – VWR, 0777-1L

Blocking solution – 0.1% PBTX or PBTW and 5% BSA in 1XPBS

Rabbit anti-FOXO – GenScript

Rabbit anti-TOR – GenScript

Goat anti-rabbit IgG AlexaFluor 488 – Invitrogen, A27034
